# Evaluation of the presence of neuropathic pain and/or central sensitization in patients with radiographic axial spondyloarthritis followed at a tertiary hospital

**DOI:** 10.1186/s42358-025-00475-2

**Published:** 2025-09-24

**Authors:** Débora de Jesus Sena, Maria Nathalia Gabriela Rocha Pontes de Oliveira, Maria Roberta Melo Pereira Soares, Luís Fábio Barbosa Botelho, Alessandra de Sousa Braz

**Affiliations:** 1 Center for Medical Sciences, Federal University of Paraiba, João Pessoa/PB, Brazil; 2 Lauro Wanderley University Hospital, Federal University of Paraiba, João Pessoa/PB, Brazil

**Keywords:** Radiographic axial spondyloarthritis, Neuropathic pain, Central sensitization, Fibromyalgia

## Abstract

**Background:**

Chronic pain (CP) in radiographic Axial Spondyloarthritis (r-axSpA) is primarily peripheral nociceptive pain. However, many patients experience persistent pain despite treatment, suggesting other underlying mechanisms, such as central sensitization (CS) and neuropathy. This study assessed the presence of CS and/or neuropathic pain (NP) in r-axSpA patients treated at a University Hospital in the state of Paraiba.

**Methods:**

This quantitative, descriptive, and cross-sectional observational study used a convenience sample collected over 24 weeks. Instruments included the Central Sensitization Inventory - CSI (positive score ≥ 40); The PainDETECT Questionnaire - PD-Q (positive score ≥ 19); clinical epidemiological questionnaires, and preliminary diagnostic for fibromyalgia (FM) [American College of Rheumatology criteria of 2010/2011, revised in 2016], applied when CS positive. Inclusion criteria: patients diagnosed with r-axSpA; aged ≥ 18. Analyses performed: Descriptive; univariate and multivariate linear regressions; Student’s T-test.

**Results:**

This study included 52 patients (34 men and 18 women), of whom 61.54% had CS. Among these, 65.62% had FM, with an average widespread pain index of 7.87. NP was present in 17.31%, and 15.38% had both CS and NP. The CSI showed a positive correlation with Bath Ankylosing Spondylitis Disease Activity Index - BASDAI (*r* = 0.574) and Axial Spondyloarthritis Disease Activity Score - ASDAS (*r* = 0.622), while the PD-Q was positively correlated with BASDAI (*r* = 0.515) and ASDAS (*r* = 0.551). In the univariate analysis, CSI was a predictor (*p* < 0.001) of both BASDAI and ASDAS, with 40.63% of patients scoring BASDAI ≥ 4 and 43.75% scoring ASDAS ≥ 2.1. Additionally, 66.67% of patients with PD-Q ≥ 19 had BASDAI ≥ 4 and 55.56% had ASDAS ≥ 2.1. The PD-Q was a predictor of BASDAI and ASDAS (*p* < 0.001).

**Conclusions:**

This study is the first in the Brazilian scientific literature to investigate and demonstrate the occurrence of CS and/or NP in patients with r-axSpA, even when adequately treated. Another finding considered relevant was the demonstration of the presence of CS in 34.38% of patients who did not meet preliminary criteria for the diagnosis of FM, emphasizing the importance of investigating other painful mechanisms in chronic inflammatory joint diseases. These findings represent a first step for future research, in larger samples and in other forms of axial spondyloarthritis.

## Introduction 

Chronic pain (CP) is a highly prevalent condition worldwide, with numerous studies highlighting the dynamic relationship between disabilities and physical, mental, and psychosocial disorders that manifest in affected individuals. Among long-term conditions, CP is responsible for the highest number of years lived with disability, leading to significant detriments not only in quality of life but also in socioeconomic aspects [1, 2].

According to the International Association for the Study of Pain (IASP), there are three main types of pain conditions: nociceptive, neuropathic, and nociplastic pain. Nociceptive pain is defined as pain related to the activation of peripheral nerve endings of primary afferent neurons, caused by noxious stimuli, whether thermal, mechanical, or chemical. From a clinical perspective, the pain response corresponds to the intensity of the nociceptive stimulus, as observed in burns or blunt trauma [3, 4]. Neuropathic pain, on the other hand, arises from injury or disease of the central nervous system (CNS) or peripheral (somatosensory). It is also considered neuropathic when the pain follows a neuroanatomical distribution consistent with the affected nervous system, or when it is supported by clinical, laboratory, and/or imaging findings [3, 4].

In contrast, nociplastic pain is defined by the IASP as “pain that arises from altered nociception despite no clear evidence of actual or threatening tissue damage, activation of peripheral nociceptors, or disease or injury of the somatosensory system that would cause the pain”. Central sensitization (CS) is considered the primary mechanism underlying nociplastic pain, characterized by an increased pain response from nociceptive neurons in response to a subliminal or normal stimulus. Thus, allodynia and hyperalgesia are indicators of nociplastic pain [2–4].

Ankylosing spondylitis (AS) is a chronic inflammatory disease classified within the group of spondyloarthritis, which primarily affects the spine and may lead to progressive stiffness and functional limitation of the axial skeleton. The Assessment of SpondyloArthritis international Society (ASAS), a global consortium of experts, reached a consensus on updates to the nomenclature and evaluation criteria related to axial spondyloarthritis (axSpA), including the terminology for diagnosis and disease activity assessment tools. One of the key decisions was that the terms AS and radiographic axial spondyloarthritis (r-axSpA) may be used interchangeably, although the preferred term is r-axSpA [5].

Patients with radiographic axial spondyloarthritis (r-axSpA), despite receiving appropriate treatment, may report persistent pain symptoms even in the absence of active inflammation typically associated with the disease. Initially, it was believed that the pain in r-axSpA stemmed exclusively from peripheral nociceptive mechanisms, with noxious stimuli being linked to the inflammatory process. However, the persistence of pain in some patients even after the resolution of inflammation suggests the involvement of neuropathic and/or nociplastic mechanisms. These may be associated with central sensitization (CS), potentially caused by somatosensory system lesions or by abnormalities in central pain processing pathways [6–8].

CS is a complex process that arises from two interconnected factors. The first is the continuous stimulation of C nerve fibers, which transmit pain signals in response to injuries or potential damage, as observed in r-axSpA-related inflammation. The second is the modulation exerted by the CNS, which can either reduce or amplify pain perception. This modulation may be impaired by dysfunctions in the mechanisms that typically inhibit pain or may be intensified by psychological factors such as depression, anxiety, stress, and negative thoughts related to pain [4, 9, 10].

Following the same pattern, neuropathic pain (NP) actively interferes with the course of pain symptoms in r-axSpA. Although the underlying pathophysiology remains insufficiently studied, NP is known to be associated with alterations in neurotransmitters, including norepinephrine, dopamine, glutamate, Gamma-AminoButyric Acid (GABA), serotonin, and others. The literature highlights that norepinephrine and serotonin contribute to sensitizing nerve endings and promoting pain perception. This action is mediated by complex molecular mechanisms involving the activation of specific receptors and the production of intracellular second messengers [11].

CS can be present in various diseases and is widely observed in patients with fibromyalgia (FM). Given the high incidence of FM in patients with CS, several authors emphasize the importance of identifying FM in this context [12, 13]. Additionally, to improve understanding, several tools have been developed to help identify and assess the presence of CS, one of which is the Central Sensitization Inventory (CSI) [6, 14]. For NP, the PainDETECT Questionnaire (PD-Q) is one of the instruments used for identification [15, 16].

Proper characterization and management of CP in patients with chronic inflammatory diseases such as r-axSpA are crucial for reducing unfavorable outcomes, preventing the misuse of medications, and avoiding treatment discontinuation. Additionally, the lack of national data on the subject motivated this study at a University Hospital in Paraiba, which aimed to assess the presence of CS and/or NP in patients with r-axSpA.

## Methodology

### Sample population and ethical aspects

A quantitative, descriptive, cross-sectional, observational study was conducted using a convenience sampling method. The research was carried out at the rheumatology department of Lauro Wanderley University Hospital (HULW), in João Pessoa, Paraiba, Brazil. The target population consisted of individuals diagnosed with r-axSpA, who were followed between October/2023 and May/2024.

The study included patients aged ≥ 18 years with a previous diagnosis of r-axSpA using the modified New York criteria (1984) [17]. Patients were excluded if they presented: non-radiographic axial spondyloarthritis; cognitive impairment preventing them from completing the research questionnaires; severe neurological comorbidities (e.g., stroke sequelae and degenerative neuropathies); psychiatric disorders associated with somatic symptoms of psychogenic origin (e.g., somatic symptom disorder and conversion disorder); people with diabetes, regardless of the duration of the disease.

The sample was chosen using a non-probabilistic method, using convenience sampling, seeking to cover the largest number of individuals. Before their medical appointments, patients were identified based on pre-established inclusion and exclusion criteria and were then invited to participate in the study. Those who agreed to take part signed the Informed Consent Form (ICF). Full confidentiality and anonymity of the collected data were ensured, and participation in the study did not interfere with the medical care provided. All participants received detailed information about the study and had the opportunity to clarify any questions.

After signing the ICF, the CSI and PD-Q instruments were administered. Based on the CSI scores, diagnostic criteria for fibromyalgia (FM) were applied to patients who scored 40 or higher. After the consultation, additional information was collected, including sociodemographic data and disease-related variables, such as the Axial Spondyloarthritis Disease Activity Score (ASDAS) and the Bath Ankylosing Spondylitis Disease Activity Index (BASDAI).

Regarding ethical aspects, this study was approved by the Medical Ethics Committee of the Center for Medical Sciences at UFPB (CAAE No. 70626623.6.0000.5183). All participants agreed to take part in the research by providing their consent through the ICF at the beginning of the study. The identity of each research volunteer was adequately protected.

### Data collection instruments

Data collection was carried out through the application of questionnaires covering sociodemographic information, details about pain and/or diseases, and treatments. To identify and characterize CS pain, the CSI was used, and the Brazilian translated version of the PD-Q was applied for NP assessment [10, 15, 16].

A clinical form developed especially for this study was used, which includes information regarding the r-axSpA, comorbidities and patient treatment, as well as the modified New York (1984) criteria [17]. Regarding the practice of physical activity, individuals who regularly performed at least 150 min of moderate-intensity physical activity, or at least 75 min of vigorous-intensity physical activity, as recommended by the World Health Organization, were considered active practitioners [18].

The most recent forms were used, which include disease activity indices – Axial Spondyloarthritis Disease Activity Score (ASDAS) and Bath Ankylosing Spondylitis Disease Activity Index (BASDAI) – in addition to sociodemographic data, specifically: age, gender, level of education, ethnicity, marital status and information related to the disease [19, 20].

The CSI is divided into two sections: the first consists of 25 questions related to the patient’s life experiences, scored from 0 (never) to 4 (always), while the second section addresses potential previously diagnosed comorbidities. The final score is obtained by summing the responses, ranging from 0 to 100 points, and is classified into subclinical (0–29), mild (30–39), moderate (40–49), severe (50–59), and extreme (60–100) levels [21, 22]. CS is considered positive when the patient scores 40 or more points on this instrument [10, 15].

The PD-Q is divided into four domains, assessing pain intensity, pain pattern over time, primary pain areas, and sensory descriptors of pain. For each question, six different response options are available, ranging from 0 (never) to 5 (very strong). By summing the scores from each domain, a total score between − 1 and 38 can be obtained. The likelihood of NP is classified as unlikely (< 13 points), uncertain (13–18 points), and probable (> 18 points) [16]. The CSI and PD-Q were administered to volunteers before or after their consultation at the rheumatology service.

The Numeric Pain Rating Scale (NPRS) was also used for grading the pain experienced by the patient at the time of the assessment, categorized as no pain (score 0), mild pain (scores 1–3), moderate pain (scores 4–6), and severe pain (scores 7–10) [23].

The preliminary diagnostic criteria for fibromyalgia (American College of Rheumatology Criteria 2010/2011 revised 2016) [24] were applied to volunteers who tested positive for CS (≥ 40 points on the CSI). This classification consists of two domains: the Widespread Pain Index (WPI), which evaluates pain distribution across different body regions, and the Symptom Severity (SS) Scale, which considers the intensity and impact of general symptoms (beyond pain) on the patient’s life. For a positive preliminary result, in addition to the persistence of symptoms for at least 3 months, one of the following scoring patterns is required: WPI ≥ 7 and SS ≥ 5, or WPI between 4 and 6 and SS ≥ 9 [13, 24]. The application of FM criteria only in participants with positive CS is justified by the close correlation between FM and CS, widely documented in the literature [12, 13]. This approach aimed to elucidate the correlation of FM with CS, since FM is prominent in patients with CS and its identification is crucial.

### Statistical analysis

Numerical variables are described using mean and standard deviation, while categorical variables are presented with absolute and relative frequencies. Pearson’s correlation coefficient was used to assess the correlation between numerical variables, and Student’s t-test was applied to compare differences between groups.

Univariate and multivariate linear regression models were used to assess the relationship between numerical variables. In the multivariate analysis, three specific models were conducted: CSIxNPRSxFM, CSIxBASDAIxFM, and CSIxASDASxFM, with FM considered a confounding factor in each analysis. The multivariate analyses were conducted to more accurately examine the associations between clinical variables and central sensitization (CSI), while controlling for potential confounding effects of fibromyalgia (FM). Given that FM may significantly influence pain scores and disease activity indices, including it as a confounding factor in the multivariate analyses performed allows for the isolation of the effects of other variables, resulting in more robust and reliable estimates of the relationships under investigation. The significance level adopted was 5%. Data analysis was performed using R software v.4.2.1.

## Results

This study included 52 patients diagnosed with r-axSpA; 34 men (65.38%) and 18 women (34.62%), with a mean age of 45 years, ranging from 20 to 72 years. Additionally, there was a predominance of mixed-race individuals (59.62%), 50% were married, and 42.31% had completed high school (Table 1).

**Table 1:** Sociodemographic characteristics of 52 patients

Variables	Absolute value	Percentage	Average	Standard deviation
**Gender**				
Men	34	65.38%		
Women	18	34.62%		
Total	52	100.00%		
**Age**			45.42	11.38
20–29	05	09.62%		
30–39	11	21.15%		
40–49	15	28.85%		
50–59	15	28.85%		
≥ 60	06	11.54%		
Total	52	100.00%		
**Ethnicity**				
White	13	25.00%		
Brown	31	59.62%		
Black	08	15.38%		
Total	52	100.00%		
**Marital Status**				
Single	18	34.62%		
Married	26	50.00%		
Stable Union	06	11.54%		
Widower	02	03.85%		
Divorced	00	0.00%		
Total	52	100.00%		
**Educational Attainment**				
Reads and writes	04	07.69%		
Incomplete primary education	15	28.85%		
Completed primary education	03	05.77%		
Incomplete high school	01	01.92%		
Completed high school	22	42.31%		
Incomplete higher education	01	01.92%		
Complete higher education	06	11.54%		
Total	52	100.00%		

The majority (59.61%) had been living with the disease for 5 to 14 years, and HLA-B27 was positive in 53.85% of patients. Regarding comorbidities (Table 2), the most prevalent was systemic arterial hypertension (SAH), affecting 36.54% of participants. Other conditions observed included osteoporosis, osteoarthritis, uveitis, and obesity. Concerning lifestyle habits, only one patient reported smoking. Thirteen patients were sedentary (25%), while 14 reported engaging in physical activities (26.92%), including weight training, swimming, pilates, cycling, or walking.

**Table 2:** Disease-related characteristics in the 52 patients

Variables	Absolute value	Percentage
**Comorbidities**		
Osteoporosis	03	05.77%
Osteoarthritis	10	19.23%
Systemic Arterial Hypertension	19	36.54%
Uveitis	11	21.15%
Obesity	03	05.77%
**Smoking**		
Active Smoker	01	01.92%
Former Smoker	07	13.46%
Never Smoked	25	48.08%
Unknown	19	36.54%
Total	52	100.00%
**Physical Activity**		
No	13	25.00%
Yes	14	26.92%
Unknown	25	48.08%
Total	52	100.00%
**Use of TNF inhibitor**		
Adalimumab	09	17.31%
Infliximab	11	21.15%
Golimumab	09	17.31%
Certolizumab	03	05.77%
Etanercept	04	07.69%
**Use of Anti-IL17**		
Secukinumab	09	17.31%
**Use of csDMARDs**		
Methotrexate ≥ 15 mg	04	07.69%
Sulfasalazine	09	17.31%
**Use of Anticonvulsants**		
Pregabalin	07	13.46%
Gabapentin	07	13.46%
**Antidepressants**		
SNRIs	04	07.69%

TNF inhibitor: Tumor necrosis factor inhibitor; Anti-IL17: Anti-interleukin 17; csDMARDs: Conventional synthetic disease-modifying anti-rheumatic drugs; SNRIs: Serotonin and norepinephrine reuptake inhibitors

Regarding the CSI analysis, 61.54% of patients with r-axSpA tested positive for CS, with an average score of 44.5 points, ranging from 5 to 97. Among these patients, 26.92% were classified as moderate, 11.54% as severe, and 23.08% as extreme (Table 3). When analyzing results by sex, 55.88% (*n* = 19) of male participants tested positive for CS, with 26.47% classified as severe or extreme. The average CSI score among men was 41.73. In contrast, 72.22% (*n* = 13) of female participants tested positive, and 50% of them were classified as severe or extreme. The average CSI score among women was 49.72. A Student’s t-test was performed to compare CSI scores between male and female participants, revealing a p-value of 0.0557. This fact shows that there is no statistically significant difference between the sexes, although this value is very close to the significance level of 0.05.

Concerning the assessment of NP using the PD-Q, 27 participants were categorized as unlikely, 16 as uncertain, and 9 as likely to present NP. The average PD-Q score was 11.61, with scores ranging from 1 to 24 points (Table 3). When stratified by sex, 58.82% of male participants were classified as unlikely for NP, and 11.76% as likely, with an average score of 10.76. Among female participants, 38.89% were unlikely, and 27.78% were likely to have NP. The average PD-Q score among women was 13.22. A Student’s t-test comparing PD-Q scores between male and female participants yielded a p-value of 0.0905, indicating no statistically significant difference between the groups. Additionally, 15.38% of the total r-axSpA patients (*n* = 8) were found to have coexisting CS and NP.

Regarding the NPRS at the time of the study, 23.08% of participants reported no pain, 25% reported mild pain, 34.62% reported moderate pain, and 17.31% reported severe pain.

**Table 3:** Distribution of r-axSpA patients according to the variables CSI, PD-Q, and NPRS

Variables	Absolute Value	Percentage	Average	Standard deviation
**CSI**			44.50	17.20
Negative for CS (< 40)	20	38.46%		
Moderate (40–49)	14	26.92%		
Severe (50–59)	06	11.54%		
Extreme (60–100)	12	23.08%		
Total	52	100.00%		
**PD-Q**			11.61	06.27
Unlikely (< 13)	27	51.92%		
Uncertain (13–18)	16	30.77%		
Probable (> 18)	09	17.31%		
Total	52	100.00%		
**NPRS**			03.69	02.70
No Pain (0)	12	23.08%		
Mild Pain (1–3)	13	25.00%		
Moderate Pain (4–6)	18	34.62%		
Severe Pain (7–10)	09	17.31%		
Total	52	100.00%		

CSI: Central Sensitization Inventory; CS: Central Sensitization; PD-Q: PainDETECT Questionnaire; NPRS: Numeric Pain Rating Scale

Regarding FM assessment (Table 4), the preliminary diagnostic criteria were applied to 32 individuals who tested positive for CS. Among them, 65.62% tested positive for FM, while 34.38% tested negative. The mean WPI score was 7.88, and the mean SS score was 7.59.

When analyzing the subset of the sample with positive CS (*n* = 32), it was found that 34.38% of these individuals had an uncertain NP result based on the PD-Q, and 25% had a probable NP result. Additionally, regarding NPRS in these individuals (Table 4), 46.88% reported moderate pain, while 25% reported severe pain. The mean NPRS score in these patients was 4.75, which was higher than that observed in the total sample of r-axSpA patients.

Analyzing the disease activity levels in patients with CS (Table 4), 40.63% of participants had a BASDAI score ≥ 4. For the ASDAS, 11 individuals (34.38%) were classified as having high disease activity, and 3 (9.38%) as having very high activity.

Analyzing the subset of participants who scored > 18 on the PD-Q, classified as likely NP (*n* = 9), it was observed that one patient did not present CS; one had moderate CS; two had severe CS; and five (55.56%) had extreme CS. The average CSI score among patients with NP was 61.88 points. Regarding disease activity, 66.67% had a BASDAI score ≥ 4, with an average of 4.93, and 55.55% had an ASDAS score ≥ 2.1, with an average of 2.84. For the NPRS, the average score among individuals with NP was 5.66, ranging from 3 to 8.

**Table 4:** Distribution of CS-positive patients by NPRS, BASDAI, ASDAS, and FM variables

Variables	Absolute value	Percentage	Average	Standard deviation
**NPRS**			4.75	2.34
No Pain (0)	03	09.38%		
Mild Pain (1–3)	06	18.75%		
Moderate Pain (4–6)	15	46.88%		
Severe Pain (7–10)	08	25.00%		
Total	32	100.00%		
**BASDAI**			3.53	2.27
< 4 points	17	53.13%		
≥ 4 points	13	40.63%		
Unknown	02	06.25%		
Total	32	100.00%		
**ASDAS**			2.49	1.01
< 1.3 (Inactive)	04	12.50%		
1.3–2.0 (Low)	03	09.38%		
2.1–3.5 (High)	11	34.38%		
> 3.5 (Very High)	03	09.38%		
Unknown	11	34.38%		
Total	32	100.00%		
**Fibromyalgia**				
Positive	21	65.62%		
Negative	11	34.38%		
Total	32	100.00%		
**WPI**			7.88	3.52
< 5 points	04	12.50%		
5–9 points	23	71.88%		
10–14 points	02	06.25%		
15–19 points	03	09.38%		
**SS**			7.59	2.63
< 5 points	05	15.63%		
5–8 points	11	34.38%		
9–12 points	16	50.00%		

PD-Q: PainDETECT Questionnaire; NPRS: Numeric Pain Rating Scale; BASDAI: Bath Ankylosing Spondylitis Disease Activity Index; ASDAS: Axial Spondyloarthritis Disease Activity Score; WPI: widespread pain index; SS: symptom severity scale

**Fig. 1:**
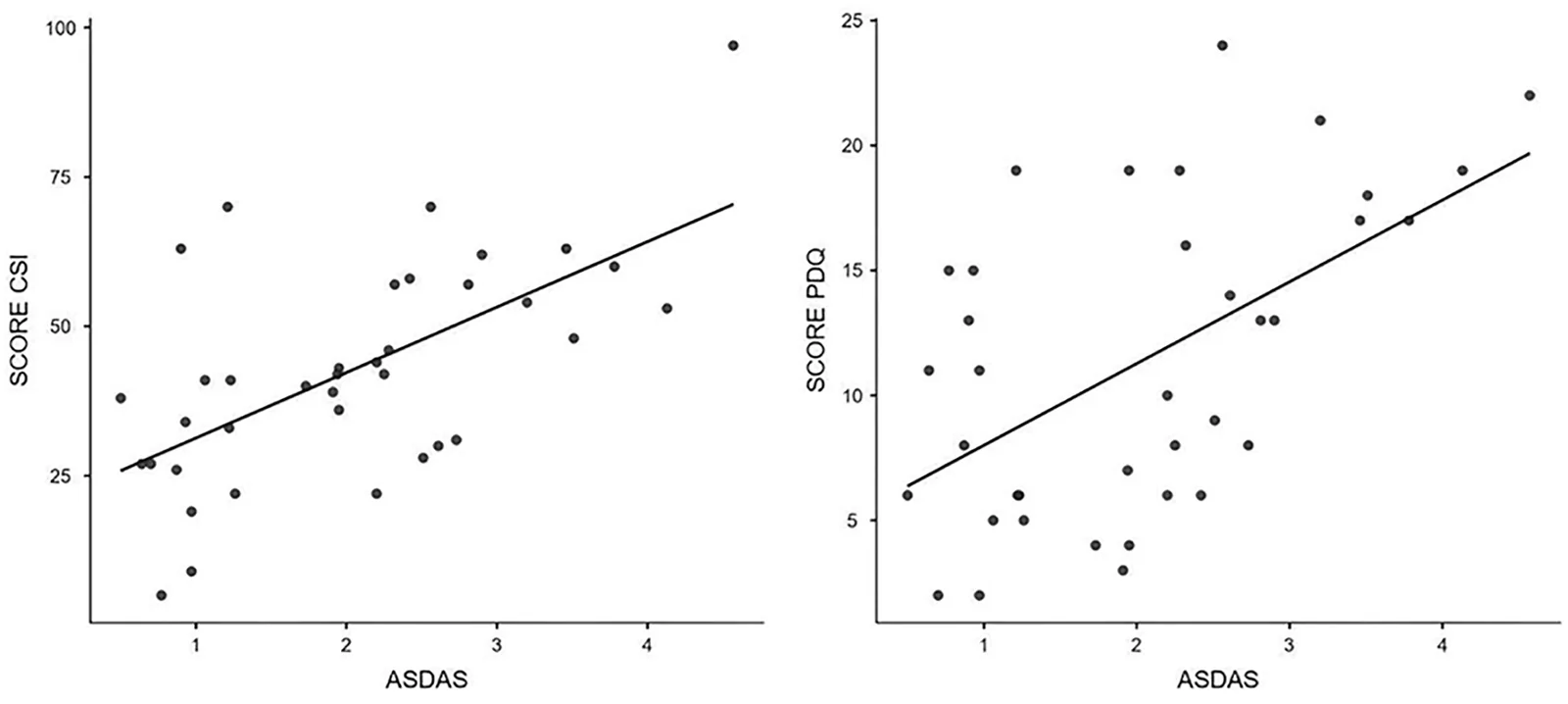
Scatter plot between the variables CSI versus ASDAS and PDQ versus ASDAS. CSI: Central Sensitization Inventory; PD-Q: PainDETECT Questionnaire; ASDAS: Axial Spondyloarthritis Disease Activity Score

Table 5 presents the main statistical analyses conducted using CSI and PD-Q as key components, including linear regression and correlation analyses. It is evident that, in univariate analysis, CSI was a predictor of NPRS and had a positive correlation (*r* = 0.580). There was no predictive relationship between CSI and physical activity (*p* = 0.744) or between CSI and smoking (*p* = 0.639). Additionally, in univariate analysis, CSI was found to be a predictor of both BASDAI and ASDAS, with positive correlations between these variables (*r* = 0.574 and *r* = 0.622, respectively), also seen in Fig. 1. No correlation was found between CSI and disease duration (*p* = 0.318, *r* = 0.144). Furthermore, after conducting a Student’s t-test analysis between HLA-B27 and CSI, no statistically significant difference was found between them (*p* = 0.309).

Multivariate analysis between CSI, NPRS, and FM diagnostic criteria showed that CSI was not a predictor of NPRS, regardless of FM presence or absence (*p* = 0.253). Likewise, in multivariate analysis between CSI, BASDAI, and FM presence, CSI was not a predictor of BASDAI, regardless of FM presence (*p* = 0.155). Finally, in multivariate analysis between CSI, ASDAS, and FM presence, CSI was not a predictor of ASDAS, regardless of FM presence (*p* = 0.214).

**Table 5:** Average score of data collection instruments by group: total sample, CS positive, and likely NP

Variables	*p*-Value¹	*R*-Multiple²	*R*-Squared²
**Univariate Analysis**			
CSI and NPRS	< 0.001	0.580	0.336
CSI and BASDAI	< 0.001	0.574	0.330
CSI and ASDAS	< 0.001	0.622	0.387
Physical Activity and CSI	0.744	0.338	
Smoking and CSI	0.639	0.084	
Diagnostic Time and CSI	0.318	0.144	
PD-Q and NPRS	< 0.001	0.494	0.244
PD-Q and BASDAI	< 0.001	0.515	0.265
PD-Q and ASDAS	< 0.001	0.551	0.304
Physical Activity and PD-Q	0.275	0.382	
Smoking and PD-Q	0.254	0.204	
Diagnostic Time and PD-Q	0.046	0.284	
**Multivariate Analysis**			
CSI, NPRS and FM	0.253	0.475	
CSI, BASDAI and FM	0.155	0.602	
CSI, ASDAS and FM	0.214	0.526	

¹= Linear regression analysis between variables. ²= Correlation analysis between variables. CSI: Central Sensitization Inventory; NPRS: Numeric Pain Rating Scale; BASDAI: Bath Ankylosing Spondylitis Disease Activity Index; ASDAS: Axial Spondyloarthritis Disease Activity Score; PD-Q: PainDETECT Questionnaire; FM: Fibromyalgia

Regarding the PD-Q, Table 5 shows that, in univariate analysis, PD-Q was a predictor of NPRS. Similarly, in univariate analysis, PD-Q was a predictor of both BASDAI and ASDAS, with positive correlations between these variables (*r* = 0.515 and *r* = 0.551, respectively), also seen in Fig. 1. It was also observed that there was no predictive relationship between smoking and PD-Q (*p* = 0.254) or between physical activity and PD-Q (*p* = 0.275). When analyzing PD-Q and disease duration, a weak negative linear correlation was found (Pearson’s *R*=-0.284) with statistical significance (*p* = 0.046). Additionally, after applying Student’s t-test between HLA-B27 and PD-Q, no statistically significant difference was found between them (*p* = 0.064).

**Fig. 2:**
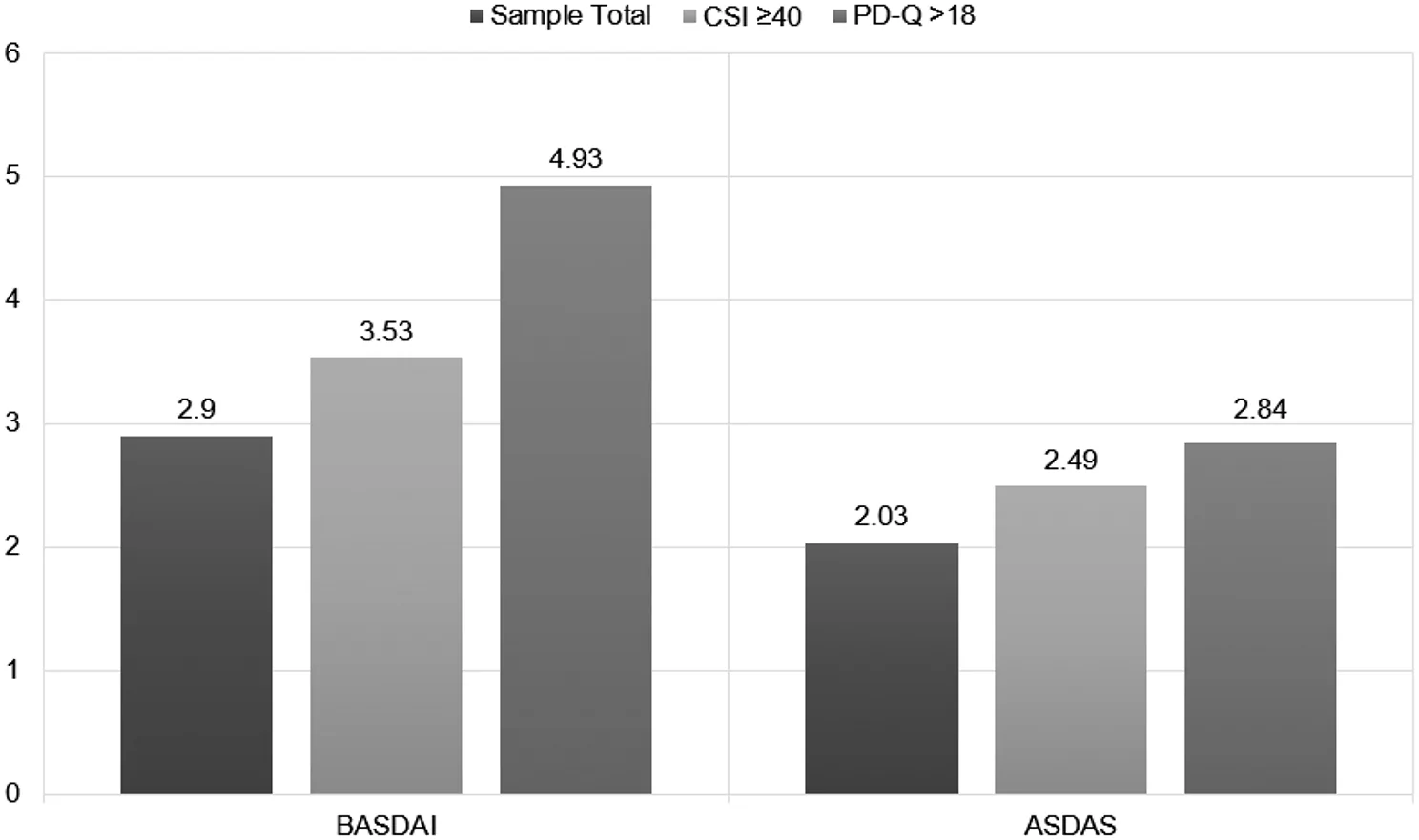
Impact of predictive CSI and PD-Q scores on BASDAI and ASDAS averages. CSI: Central Sensitization Inventory; PD-Q: PainDETECT Questionnaire; BASDAI: Bath Ankylosing Spondylitis Disease Activity Index; ASDAS: Axial Spondyloarthritis Disease Activity Score

Figure 2 presents the average scores for BASDAI and ASDAS across different participant groups: the total sample, those with positive CS, and those with probable NP. When comparing the total sample to the CS-positive group, the mean BASDAI score was 2.90 vs. 3.53, and the mean ASDAS score was 2.03 vs. 2.49, respectively. When comparing the total sample to the NP-positive group, the mean BASDAI score was 2.90 vs. 4.93, and the mean ASDAS score was 2.03 vs. 2.84, respectively.

## Discussion

The main sociodemographic characteristics of this study included: predominance of male participants, an average age of 45 years, mixed-race individuals, married participants, and those with a high school education. The average time since diagnosis was approximately 9 years, and more than half of the sample tested positive for HLA-B27. These findings are consistent with the literature, which reports that r-axSpA has a high incidence among young male populations, particularly in individuals carrying the HLA-B27 marker, present in 60% of r-axSpA cases in Brazil [25–27].

Regarding comorbidities found in patients, the presence of uveitis stands out, a phenomenon well described in the work of Citera et al. [28], who found that this extra-articular manifestation was the most commonly reported, appearing in 20 of the 41 scientific publications he analyzed.

In terms of pharmacological treatment for r-axSpA, the literature extensively describes that the mainstay of treatment includes nonsteroidal anti-inflammatory drugs (NSAIDs) and biologics such as TNF-α inhibitors or anti-IL17 agents (secukinumab). The use of csDMARDs, such as methotrexate and sulfasalazine, can help reduce symptoms. When patients continue to present high disease activity (ASDAS ≥ 2.1 and/or BASDAI ≥ 4) for more than 12 weeks after using TNF inhibitors and/or anti-IL 17, in addition to reevaluating the diagnosis, it is recommended to switch to another biological medication and add/change NSAIDs [25, 29]. Additionally, medications for CP management can contribute to reducing or even eliminating pain, including antidepressants and/or anticonvulsants such as pregabalin or gabapentin [30].

Following these treatment guidelines, the management of r-axSpA patients in this sample appeared to be appropriate. However, despite this, the mean NPRS score in this study remained around 4, and the mean disease activity indices (BASDAI and ASDAS) remained elevated. These findings may be related to the presence of CS and/or NP, observed in approximately 64% of patients. Thus, conventional r-axSpA treatment may not have provided adequate pain relief. In the studies by Zhou et al. [11], it was found that the use of TNF inhibitor drugs was ineffective in improving NP associated with r-axSpA. The literature supports the use of both pharmacological and non-pharmacological therapies to attempt to reduce CS; however, none of these approaches have demonstrated significant effects on the characteristics of this pathology [13].

Regarding CS, a study with 182 participants conducted by Kieskamp et al. [10] showed that CS negatively impacted the quality of life of r-axSpA patients, as measured by the Ankylosing Spondylitis Quality of Life Questionnaire (ASQoL). Furthermore, the study observed that CSI scores were strongly associated with high disease activity. When comparing individuals with CSI ≥ 40 to those with CSI < 40 in these same studies, it was found that the first group had higher ASDAS scores (mean 2.6 vs. 1.7), higher BASDAI scores (mean 5.2 vs. 2.8), and higher ASQoL scores (mean 9.7 vs. 3.3), respectively. Similar results were observed in studies by Sariyildiz et al. [31] as well as Koca, Göğebakan, and Çetin [32] on these relationships.

In accordance with the literature, the univariate analyses between the CSI and disease activity indices in this study showed a correlation and predictive relationship between these variables. These findings reinforce the understanding that the presence of CS should be considered in individuals with r-axSpA who continue to experience pain despite optimized treatment, as it significantly impacts their quality of life.

When applying the preliminary criteria for FM, it was found that approximately 35% of individuals with CS did not meet the FM diagnostic criteria used, demonstrating that FM was not associated with the presence of CS in a significant portion of patients.

However, in multivariate analyses between CSI and BASDAI, as well as CSI and ASDAS, both using FM as a confounding factor, no predictive relationship was observed between these variables. This finding may be related to the sample size of this study, which should be reassessed in future research. Additionally, no other studies were found that conducted multivariate analyses between these variables, except for some with partial similarities, such as the study by Sariyildiz et al. [31]. In that study, which included 108 patients with axial spondyloarthritis, a positive FM diagnosis was an important exclusion criterion. In the multiple regression analysis results, BASDAI and CS were found to behave as predictive factors.

In the results of this study, it was observed that the number of individuals with probable or uncertain NP accounted for 48.07% of the total sample. Additionally, the mean scores for NPRS and disease activity indices (BASDAI and ASDAS) were higher than those observed in the overall study sample. Similarly, some scientific studies have identified correlations between NP and BASDAI [32–34]. The study conducted by Gok et al. [35] with 128 r-axSpA patients demonstrated that the group of individuals with NP diagnosed by PD-Q had higher disease activity indices compared to those without NP - mean BASDAI of 5.42 vs. 4.0 and mean ASDAS of 3.17 vs. 2.71, respectively. It was also observed that NP is associated with greater loss of functional capacity and a decline in overall health status. Supporting these findings, the linear regression statistical analyses in this study showed that PD-Q was a predictor of disease activity indices (BASDAI and ASDAS) and had a positive correlation with these variables.

The work of Geler-Külcü et al. [34] provides data that corroborate these results. In their studies, a total of 100 patients with r-axSpA participated, being divided into two groups: group 1 – individuals with PD-Q ≥ 13 points (uncertain and probable NP), and group 2 – individuals with PD-Q < 13 points (unlikely NP). Disease activity was analyzed using BASDAI, ASDAS, Maastricht Ankylosing Spondylitis Enthesitis Score (MASES) and Patient Global Assessment of Disease Activity (PGA), as well as functional level using the Bath Ankylosing Spondylitis Functional Index (BASFI) and health-related quality of life (36-Item Short Form Survey (SF-36). It was seen that the means of the variables BASDAI (5.0 vs. 3.2), PGA (6.0 vs. 4.0), ASDAS (3.2 vs. 2.4) and BASFI (5.4 vs. 2.05) were significantly higher in group 1 compared to group 2, as well as the physical component score of the SF-36 was significantly lower in group 1 compared to group 2 (31.9 vs. 41.3), evidencing a great impact of NP on the prognosis of patients with r-axSpA.

The analyses of this study revealed that female participants scored higher on both the CSI and the PD-Q compared to male participants, although there was no statistical significance in the group analyses. Comparing these findings with the literature, some studies report discrepancies regarding the statistical influence of sex on CS or NP [10, 31, 32, 33]. However, the studies by Kedyk and Stanislavchuk [33] found that in their sample (*n* = 142), the prevalence of NP was 51.7% in women and 32.7% in men. Similarly, in the study by Kieskamp et al. [10], high CSI scores were significantly more frequent in women (60% vs. 29%).

Regarding participants who tested positive for both CS and NP, the studies by Guler, Celik, and Ayhan [36] elucidate that the overlap of these two conditions may result in worsened chronic pain associated with r-axSpA, suggesting that patients with this coexistence require individualized care.

Some limitations should be noted in this research, including the sample size, which may have affected, among other aspects, the results of the multivariate statistical analyses and the analysis by group of the mean scores on the CSI and PD-Q, data that could be better investigated in future studies.

This study included only patients with axial spondyloarthritis with radiographic evidence of sacroiliitis (r-axSpA), based on the modified New York criteria (1984), which ensured diagnostic homogeneity of the sample. However, this approach limits the generalizability of the findings to patients with the non-radiographic form of the disease, whose exclusion was deliberate due to the clinical focus of the study and the diagnostic criteria used at the reference institution where the study was conducted.

In addition, the participants’ recall factor when answering the questionnaires may have influenced their responses and the final scores. The small number of scientific studies on this topic in the main databases makes it difficult to compare the results obtained with other studies, validate the conclusions and generalize the findings to a wider population.

## Conclusion

The presence of CS and/or NP was observed in more than 60% of r-axSpA patients, and among those who tested positive for CS, 34.38% did not meet the preliminary criteria for FM. These conditions were significant contributors to increased pain intensity reported by patients and higher disease activity indices, as measured by BASDAI and ASDAS. Both CS and NP should be considered in r-axSpA patients who continue to experience pain despite optimized treatment for CP and r-axSpA, including in the absence of FM.

Despite the limitations, this research has significant importance in the Brazilian scientific literature by investigating, for the first time, the presence of CS and/or NP in patients with r-axSpA — especially given the lack of similar Brazilian studies addressing this issue. In this sense, our findings represent a first step for future research.

## Data Availability

No datasets were generated or analysed during the current study.

## Abbreviations

Anti-IL 17: Anti-interleukin 17
Anti-TNF: Anti-tumor necrosis factor
ASDAS: Axial Spondyloarthritis Disease Activity Score
ASQoL: Ankylosing Spondylitis Quality of Life Questionnaire
BASDAI: Bath Ankylosing Spondylitis Disease Activity Index
BASFI: Bath Ankylosing Spondylitis Functional Index
CP: Chronic Pain
CS: Central Sensitization
CSI: Central Sensitization Inventory
csDMARDs: Conventional Synthetic Disease-Modifying Antirheumatic Drugs
FM: Fibromyalgia
HLA-B27: Human Leukocyte Antigen B27
MASES: Maastricht Ankylosing Spondylitis Entesitis Score
NP: Neuropathic Pain
NPRS: Numeric Pain Rating Scale
PD-Q: PainDETECT Questionnaire
PGA: Patient Global Assessment of Disease Activity
r-axSpA: Radiographic Axial Spondyloarthritis
SAH: Systemic Arterial Hypertension
SF-36: 36-Item Short Form Survey
SNRIs: Serotonin and Norepinephrine Reuptake Inhibitors
SS: Symptom Severity Scale
WPI: Widespread Pain Index
